# Release of Neuronal HMGB1 by Ethanol through Decreased HDAC Activity Activates Brain Neuroimmune Signaling

**DOI:** 10.1371/journal.pone.0087915

**Published:** 2014-02-14

**Authors:** Jian Y. Zou, Fulton T. Crews

**Affiliations:** Bowles Center For Alcohol Studies, University of North Carolina at Chapel Hill, Chapel Hill, North Carolina, United States of America; Virginia Commonwealth University, United States of America

## Abstract

Neuroimmune gene induction is involved in many brain pathologies including addiction. Although increased expression of proinflammatory cytokines has been found in ethanol-treated mouse brain and rat brain slice cultures as well as in post-mortem human alcoholic brain, the mechanisms remain elusive. High-mobility group box 1 (HMGB1) protein is a nuclear protein that has endogenous cytokine-like activity. We previously found increased HMGB1 in post-mortem alcoholic human brain as well as in ethanol treated mice and rat brain slice cultures. The present study investigated the mechanisms for ethanol-induced release of HMGB1 and neuroimmune activation in a model of rat hippocampal-entorhinal cortex (HEC) brain slice cultures. Ethanol exposure triggered dose-dependent HMGB1 release, predominantly from neuronal cells. Inhibitors of histone deacetylases (HDACs) promoted nucleocytoplasmic mobilization of HDAC1/4 and HMGB1 resulting in increased total HMGB1 and acetylated HMGB1 release. Similarly, ethanol treatment was found to induce the translocation of HDAC1/4 and HMGB1 proteins from nuclear to cytosolic fractions. Furthermore, ethanol treatment reduced HDAC1/4 mRNA and increased acetylated HMGB1 release into the media. These results suggest decreased HDAC activity may be critical in regulating acetylated HMGB1 release from neurons in response to ethanol. Ethanol and HMGB1 treatment increased mRNA expression of proinflammatory cytokines TNFα and IL-1β as well as toll-like receptor 4 (TLR4). Targeting HMGB1 or microglial TLR4 by using siRNAs to HMGB1 and TLR4, HMGB1 neutralizing antibody, HMGB1 inhibitor glycyrrhizin and TLR4 antagonist as well as inhibitor of microglial activation all blocked ethanol-induced expression of proinflammatory cytokines TNFα and IL-1β. These results support the hypothesis that ethanol alters HDACs that regulate HMGB1 release and that danger signal HMGB1 as endogenous ligand for TLR4 mediates ethanol-induced brain neuroimmune signaling through activation of microglial TLR4. These findings provide new therapeutic targets for brain neuroimmune activation and alcoholism.

## Introduction

Neuroimmune activation in brain has been hypothesized to contribute to brain damage and behavioral changes associated with alcohol consumption. In recent years, many studies have reported that chronic alcohol consumption can increase proinflammatory cytokines and innate immune gene expression in the brain [1], [2]. Increased cytokines and other neuroimmune genes have been reported in human post-mortem alcoholic brain [3], [4], as well as following ethanol treatment of animals [5], [6] and brain slice cultures [4], [7]. Recent studies suggest activation of brain neuroimmune signaling induces changes in mood and drinking behavior and increases risk of alcoholism as well as alcoholic neurodegeneration [1]. Genetic analysis of ethanol preferring rats and mice reveals increased expression of multiple innate immune genes associated with preferring to drink ethanol [8]. Further, studies have demonstrated that Toll-like receptor 4 (TLR4) is critical for ethanol-induced neuroimmune activation, neurodegeneration and behavioral pathology [2], [6]. Treatment of mice with classic TLR4 ligand lipopolysaccharide (LPS) shows an increase in ethanol consumption and preference that persists for months [9] consistent with the prolonged brain neuroimmune response following LPS treatment of mice [10]. Central amygdala infusion of a TLR4 siRNA vector (pHSVsiLTLR4a) also inhibited binge drinking in rats [11]. Recent studies support the hypothesis that high mobility group box 1 (HMGB1) protein, an endogenous cytokine that can activate toll-like receptors including TLR4, is linked to ethanol-induced increase in expression of brain neuroimmune genes [12]. Therefore, it is conceivable that ethanol exposure may trigger release of endogenous TLR4 ligand HMGB1 contributing to ethanol-induced neuroimmune signaling through TLR4 receptor activation.

Release of HMGB1 can occur as an active process stimulated by cellular signaling processes or as a result of cell death. The release of HMGB1 by dying cells is thought to drive the necrotic cell death inflammatory response [13], [14], [15]. Active release of HMGB1 involves receptor signaling without cell death and has been studied primarily in immune cells such as monocytes [16], [17] and in hepatocytes [18]. Receptor stimulated release of HMGB1 involves acetylation that regulates nuclear and cytoplasmic levels of HMGB1 apparently through actions on nuclear enzymes that regulate protein acetylation, e.g. histone deacetylases (HDAC) and histone acetylases (HAT) [18], [19]. Active cellular HMGB1 release involves migration from the nucleus to lysosome-like vesicles that protect HMGB1 from proteolysis in the cytoplasm [16], [18]. Calcium/calmodulin-dependent protein kinase (CaMK) in monocytes activates HMGB1 migration to cytosolic vesicles and triggers exocytosis of vesicles releasing HMGB1 into the extracellular space [20], [21]. Recent studies have suggested that brain HMGB1 is highly expressed in neurons and is released by neurons [12], [22], [23], [24]. These findings are consistent with brain releasing HMGB1 that impacts neuronal signaling.

To investigate HMGB1 release in brain in response to ethanol exposure we used an *ex vivo* hippocampal-entorhinal cortex (HEC) brain slice culture model that contains all brain cell types and maintains the morphology and local circuits that could contribute to signaling. We report here that HMGB1 appears to be highly expressed in mature and immature neurons in HEC slices. Ethanol exposure triggered release of HMGB1 into the culture media and released HMGB1 was primarily acetylated HMGB1. Both ethanol and HDAC inhibitors increased HMGB1 and HDAC in cytosolic fractions and reduced levels in nuclear fractions suggesting that HDAC inhibition mobilizes protein migration from nuclear to cytoplasmic compartments. We found that ethanol decreased HDAC expression and increased acetylated HMGB1, consistent with HDAC regulating HMGB1 release in brain. Ethanol treatment induced proinflammatory cytokine genes as well as HMGB1 and TLR receptors. Targeting HMGB1 and/or TLR4 receptors blocked ethanol-induced neuroimmune activation supporting the hypothesis that ethanol induces HMGB1 release, activating TLR4-mediated neuroimmune signaling. These studies suggest that brain HDACs are linked to HMGB1-TLR4 neuroimmune signaling. The release of HMGB1 by ethanol and/or other factors provides new therapeutic targets for brain protection against neuroimmune induced degeneration and alcoholism.

## Materials and Methods

### Ethics Statement

All protocols followed in this study were carried out in strict accordance with the recommendations in the Guide for the Care and Use of Laboratory Animals of the National Institutes of Health. The animal protocol was approved by the Institutional Animal Care and Use Committee (IACUC) at University of North Carolina at Chapel Hill (IACUC ID: 13-050). All efforts were made to minimize suffering.

### Hippocampal-entorhinal Cortical Slice Culture

Organotypic hippocampal-entorhinal cortical (HEC) slice cultures were prepared as described previously [25]. Briefly, Slices were placed onto a 30 mm diameter membrane tissue insert and cultured with medium containing 75% MEM with 25 mM HEPES and Hank’s salts +25% horse serum (HS) +5.5 g/L glucose +2 mM L-glutamine in a humidified 5% CO_2_ incubator at 36.5°C for 7 days *in vitro* (DIV), followed by 4 DIV in medium containing 12.5% HS and then in serum-free medium supplemented with N2 till the end of experiment. The cultures after 14 DIV were used for experiments. Drug treatments were done during the final 4–7 DIV in serum-free N2 medium.

### Drug and Ethanol Treatments

All drug treatments were performed in serum-free N2 supplemented medium. Ethanol treatment with the indicated concentrations occurred in a desiccator containing 300 ml water saturated with equal concentrations of ethanol to balance evaporation of ethanol from the media. HMGB1 was purchased from Invitron (Invitrogen, CA) or Sigma (St. Louis, MO) and only one batch from Sigma (Cat#H4652, Lot#090M4056) show potent inflammatory activity. At the end of the experiments, media were collected and slices were removed for further analysis.

### ELISA Measurements of HMGB1

Media HMGB1 levels from each experiment were determined with ELISA kit (IBL, Germany) according to manufacturer’s instruction. Briefly, total of 50 µl of culture medium from each sample was used for ELISA. All samples were run in triplicate.

### Confocal Analysis

At the end of experiments, slice cultures were removed and fixed with 4% paraformaldehyde+5% sucrose in 0.01 M PBS and placed in cold room overnight. Free-floating slices were used for double immunofluorescent staining. Specific cellular markers used include anti-neuronal-specific nuclear protein (NeuN) for neurons, Iba-1 for microglia and GFAP for astrocytes. All primary antibodies were incubated for 48 hrs at 4°C. Either Alexa Fluor 594 or Alexa Fluor 488 secondary antibodies (1∶2000; Molecular Probes, Eugene, OR) were used for immunofluorescent staining and incubated for 1 hr at room temperature. The slices were cover slipped with anti-fade mounting medium (pro-long; Molecular Probes). Confocal analysis was performed using a LeicaSP2 AOBS Upright Laser Scanning Confocal in the Michael Hooker Microscopy Facility, UNC.

### Western Blotting

Cell lysates were prepared from the entire HEC slices. Briefly, HEC slices were incubated in lysis buffer (10 mM HEPES, 1.5 mM MgCl_2_, 10 mM KCl, pH7.9) plus protease inhibitor cocktail (Sigma) for 15 min then disrupted with sonication (3 time, 2 min apart in ice). After centrifugation of the slice homogenate, the supernatant were collected. For preparation of cytosolic and nuclear fraction of protein extracts, the pellet was disrupted again in an extraction buffer and the supernatants of nuclear fraction were collected after centrifugation. The protein concentrations were determined by using the Bio-Rad Protein Assay (Bio-Rad). For Western blotting, an equal amount of protein was mixed with 10 µl 5x loading buffer, and separated using a 4–15% Tris mini-gel (Bio-Rad), then transferred onto a PVDF membrane. After blocking with LI-COR blocking buffer overnight, the membrane was probed with mouse anti-HDAC1 (Santa Cruz, 1∶200) and rabbit anti-HDAC4 Abcam, 1∶250) at 4°C overnight. After washing, membrane was incubated with goat anti-mouse and anti-rabbit second antibodies coupled with green or red fluorescence from LI-COR Bioscience then scanned with the Odyssey machine (Lincoln, NE). The membranes were then stripped with Newblot PVDF stripping buffer (LI-COR) and re-probed with rabbit anti-HMGB1antibody (1∶500, Abcam) and mouse anti-β-actin (1∶500, Santa Cruz) at 4°C overnight. The membranes were then detected with the same secondary antibodies as mentioned above.

### Co-immunoprecipitation

Immunoprecipitation was performed to detect the status of acetylated HMGB1 in culture medium using a catch and release kit (Millipore, USA) according to the manufacturer’s instructions. Briefly, a total 250 µl of culture medium from each sample was incubated with 5 µg of antibodies against acetyl-lysine (Cell Signaling, USA) and 10 µl affinity ligand for 1 hr at room temperature. Proteins were eluted in its native form and subjected to Western blot analysis. The blots were probed with rabbit anti-HMGB1 (1∶500, Abcam).

### TLR4 or HMGB1 Knockdown with siRNAs

Rat TLR4 and HMGB1 siRNA as well as negative control siRNA were purchased from Ambion (Ambion, Grand Island, NY). The protocol of transfection in HEC slices has been described previously [26]. Briefly, the transfection mixture was added to serum-free N2 medium at a final concentration of 20 nM siRNA +6 µl Lipofectamine 2000 to a total volume of 1.2 ml(600 µl on top of the slices and 600 µl at bottom of the slice culture). Vehicle controls were treated with the same N2 medium containing negative control siRNA. After transfection for 24 hrs, siRNA-containing medium was replaced with regular serum-free N2-supplemented medium and the slices were cultured in the absence or presence of ethanol (100 mM) for 4 days. At the end of the experiments the slices were removed for real-time PCR analysis.

### RNA Isolation, Reverse Transcription and Real Time Quantitative RT-PCR

For each specific experiment, the slices were removed at the end of the experiments for purification of total RNA using the RNeasy Mini Kit (Qiagen Inc., CA). The total amount of RNA was quantified by spectrophotometry at 260 nm. For reverse transcription, 2 µg of RNA was used to synthesize the first strand of cDNA using random primers (Invitrogen) and reverse transcriptase Moloney murine leukemia virus (Invitrogen). After a 1∶2 dilution with water, 2 µl of the first strand cDNA solution was used for RT-PCR. The primer sequences for real time RT-PCR were designed by Integrated DNA Technologies (Coralville, IA) and listed in Table 1. SYBER Green Supermix (AB system, UK) was used as a RT-PCR solution. The real time RT-PCR was run with initial activation for 10 min at 95°C and followed by 40 cycles of denaturation (95°C, 40 s) annealing (58°C, 45 s) and extension (72°C, 40 s). All experiments were run in triplicate. The threshold cycle (*C*_T_) of each target product was determined and normalized to internal standard β-actin.

**Table 1:** Primer sequences for quantitative PCR analysis.

Gene	Forward primer (5′-3′)	Reverse primer (5′-3′)
TNFα	AGCCCTGGTATGAGCCCATGTA	CCGGACTCCGTGATGTCTAAG
IL-1β	GAAACAGCAATGGTCGGGAC	AAGACACGGGTTCCATGGTG
MCP-1	CCATCCGAGGAGGCCAATAC	CAAGCAGGCGATACCCAGC
MMP-9	AAGCCTTGGTGTGGCACGAC	TGGAAATACGCAGGGTTTGC
TLR2	GGAGACTCTGGAAGCAGGTG	CGCCTAAGAGCAGGATCAAC
TLR4	CCAGAGCCGTTGGTGTATCT	TCAAGGCTTTTCCATCCAAC
NALP1	GGACCAGAATCCTGAGCTGTGT	GAAGCCTCAGGAAGGATGGAT
MyD88	GACTGCCAGAAATACATACG	ATCTCCTGCACAAACTCAA
NF-κB	GGCAGCACTCCTTATCAA	GGTGTCGTCCCATCGTAG
HDAC1	CATGCCAAGTGTGTGGAGTTCGT	GTCCAGCACCGAGCGACATT
HDAC4	ATCAGAGACCCAATGCCAAT	AGCAGGTTTGACGCCTACAG
HMGB1	ATGGGCAAAGGAGATCCTA	ATTCTCATCATCTCTTCT
β-Actin	CTACAATGAGCTGCGTGTGGC	CAGGTCCAGACGCAGGATGGC

### Statistical Analysis

Data are presented as a mean ± S.E.M values from the indicated number of slice preparations in an experiment. Statistical comparisons were made with ANOVA and the difference between the experimental groups was further compared by using post hoc Fisher PLSD test. In cases with a low N, SAS permutation tests were used to determine to confirm ANOVA significance.

## Results

### Ethanol Release of HMGB1 from HEC Brain Slice Cultures

**Figure 1:**
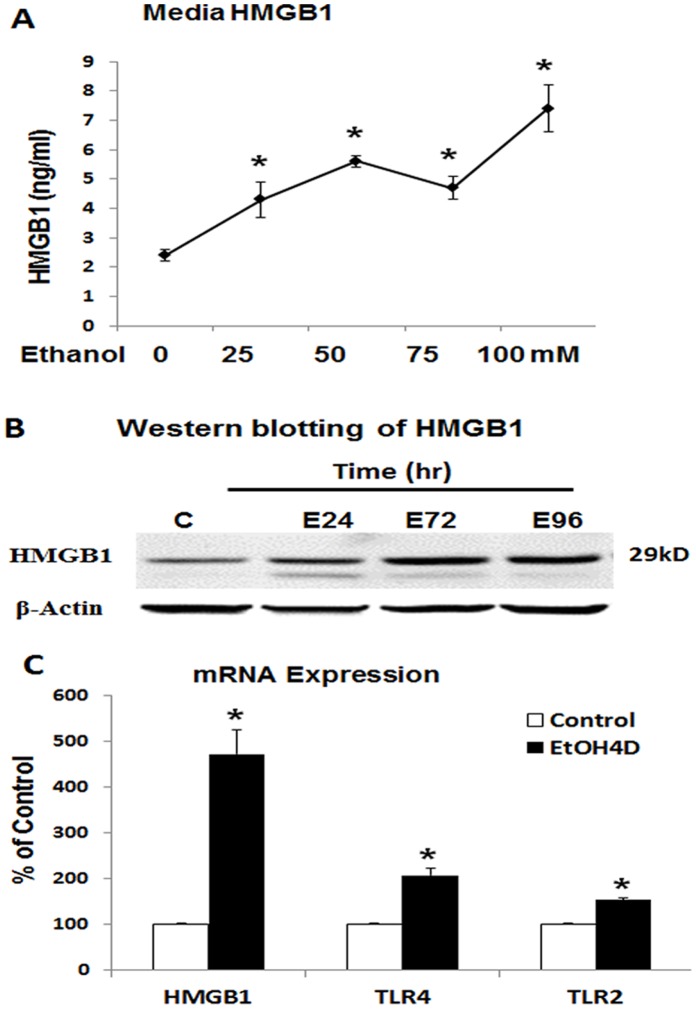
Ethanol increases HMGB1 expression and release. A: ELISA measurements indicate ethanol dose-dependently increase HMGB1 release into culture medium compared with Control (*p<0.05; n = 3); B: Western blotting of the whole cell lysate show HMGB1 protein level was increased progressively over time period in response to ethanol; C: RT-PCR analysis show ethanol-induced mRNA expression and HMGB1 as well as TLR4 and TLR2 (*p<0.05 compared with Control; n = 3).

To gain insight into the mechanisms of ethanol induced HMGB1 release, HEC slices were treated with various ethanol concentrations. Ethanol concentration response curves find a significant increase in media HMGB1 in a concentration dependent manner that at the highest concentration studied, a high binge drinking concentration, increased media HMGB1 about 4 fold (Figure 1A). To determine if cell death contributed to ethanol induced HMGB1 release we used propidium iodide (PI), a sensitive marker of cell death. Ethanol (100 mM) after 4 days did not alter PI intensity (PI intensity: 22.9±2.6 control; 26.3±3.1 ethanol; n = 8), suggesting cellular HMGB1 release in the absence of cell death. A time course of ethanol (100 mM)-induced HMGB1 release finds significant increases in media at 4 hrs that progressively increase over 4 days of exposure (data not shown). Western blot analysis of whole HEC slice lysates find ethanol treatment progressively increases the amount of HMGB1 protein (Figure 1B). In addition, ethanol treatment increased mRNA of HMGB1 as well as the HMGB1 responsive receptors, TLR4 and TLR2 (Figure 1C). These findings indicate ethanol increases HMGB1 protein and mRNA in HEC brain slices and increases media HMGB1 independent of cell death consistent with active release of HMGB1.

### Predominant Neuronal Expression and Release of HMGB1 in HEC Slice

**Figure 2:**
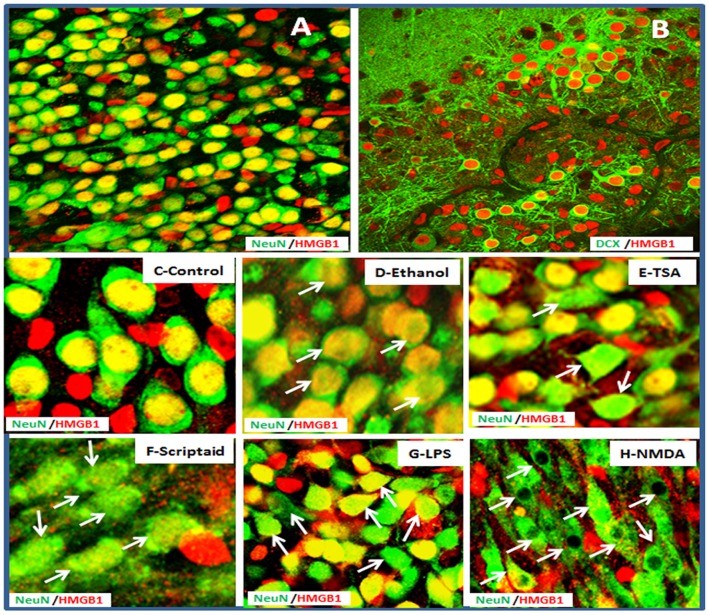
Neuronal expression of HMGB1. Representative confocal merger images showing A: double immunofluorescent staining with anti-HMGB1 (red) and anti-NeuN (green), a marker for mature neurons. Majority of NeuN+ neurons coexpressing HMGB1 (yellow); B: double immunofluorescent staining with anti-HMGB1 (red) and anti-doubleCortin (DCX, green), a marker for immature neurons. HMGB1 is located in the nuclear of majority DCX+ neurons. Mobilization and translocation of nuclear HMGB1 in HEC slices was further depicted from treatment of Control (C), ethanol (D), TSA (E), Scriptaid (F), LPS (G) and NMDA (H). Nuclear mobilization and/or cytoplasm translocation in neurons were indicated by arrows (original magnification 80x in A–B: 320x in C–H).

To investigate the cell type expression of HMGB1 in HEC brain slice cultures, we performed double immunofluorescent staining and confocal analysis using cell specific markers, NeuN for mature neurons; doubleCortin (DCX) for immature neurons; GFAP for astrocytes; and Iba-1 for microglial cells. As shown in Figure 1, HMGB1+ staining in HEC brain slice cultures is detected in nuclei of NeuN+ mature neuronal cell (94.7%±1.8% in total 1824 NeuN+ cells counted), similar to histochemical findings in mouse and postmortem human brain [12]. Interestingly, we also found HMGB1 colocalized with DCX+ immature neurons (96.5%±2.1 in total 1086 DCX+ cells counted) (Figure 2B). DCX is generally considered to be a marker for neuroprogenitors of hippocampal dentate gyrus neuroprogenitors in brain [27] as well as in this model [4]. To our surprise, HMGB1 rarely co-localized with Iba-1+ microglial cells (data not shown). HMGB1 appears to express in the nuclei of some astrocyte GFAP+ astrocytes (data not shown). These data suggest HMGB1 is highly expressed in neurons and neuroprogenitors in HEC brain slice cultures. Neuronal release of HMGB1 is further examined with double immunofluorescent staining and confocal analysis from HEC slices treated with ethanol, LPS, NMDA and HDAC inhibitors TSA and Scriptaid. Ethanol treatment decreased the intensity of nuclear HMGB1 staining with no other visible differences in neurons. Depending on the degree of insult, different stages of nuclear HMGB1 mobilization in hippocampal CA neurons were observed, from mobilization without cell death in ethanol and TSA treatments (D–E, G) to complete depletion of nuclear HMGB1 in dying neurons treated with Scriptaid (F) and NMDA (H). These results suggest predominant neuronal source of HMGB1 in this model.

### Inhibition of HDACs Releases HMGB1

**Figure 3:**
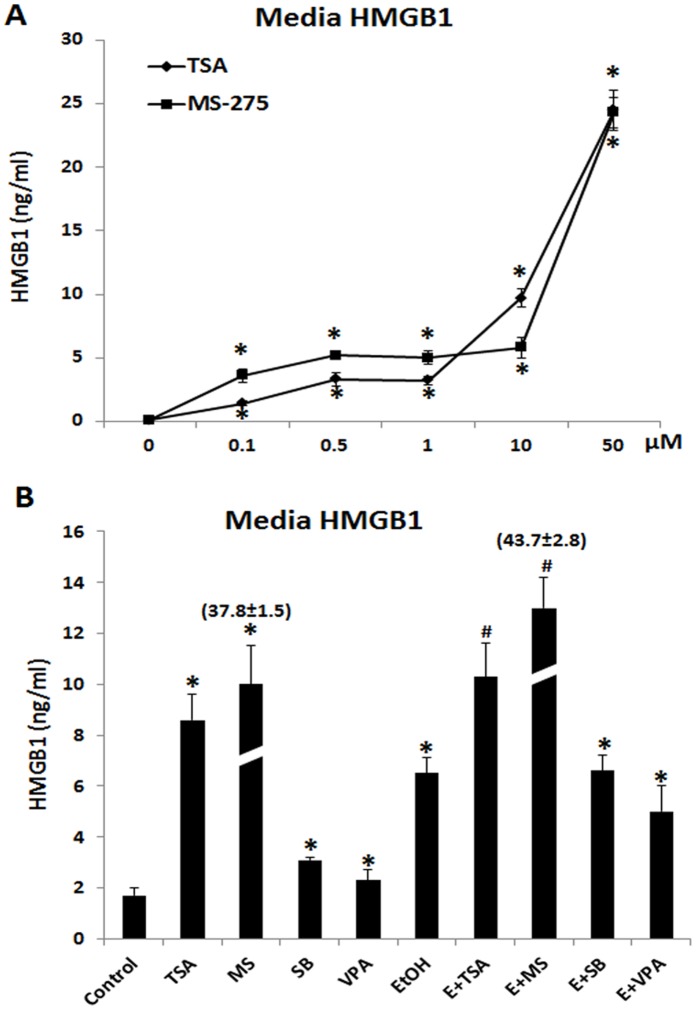
HDAC inhibitors increase HMGB1 release. A: Shown are mean ± SEM of media HMGB1 levels from a representative dose dependent study indicating broad HDAC inhibitor TSA and selective HDAC1 inhibitor MS-275 trigger active release of neuronal HMGB1 into medium (*p<0.01 compared with control; n = 3). C: ELISA measurements of media HMGB1 from cultures treated with various HDAC inhibitors alone or co-presence of ethanol. Treatments of HEC slices for 4 days with ethanol in the absence or presence of inhibitors including TSA (1 µM), MS-275 (5 µM), sodium butylate (SB, 100 µM) and vaproic acid (VPA, 100 µM) increase media HMGB1 level but only TSA and MS-275 appears to potentiate ethanol action (*p<0.01 compared with Control; #p<0.001 compared with EtOH. n = 3).

In monocytes [19], [21] and hepatocytes [18] HMGB1 synthesis and release increase simultaneously with increased HMGB1 acetylation. To determine if inhibition of HDAC impacts HMGB1 release HEC slices were treated with HDAC inhibitors trichostatin A (TSA), sodium butyrate (SB), valproic acid (VPA) and MS-275. All HDAC inhibitors increased release of HMGB1 (Figure 3). TSA, a pan-HDAC inhibitor, as well as MS-275, a specific HDAC1 inhibitor, increased HMGB1 release in a concentration dependent manner (Figure 3A). Ethanol alone increased HMGB1 release, however, combinations of HDAC inhibitors with ethanol were less than additive consistent with shared mechanisms of HMGB1 release (Figure 3B). These findings are consistent with HDAC regulating HMGB1 acetylation and release in brain.

### HDACs Alter Nuclear-cytosol Distribution and Release of HMGB1

**Figure 4:**
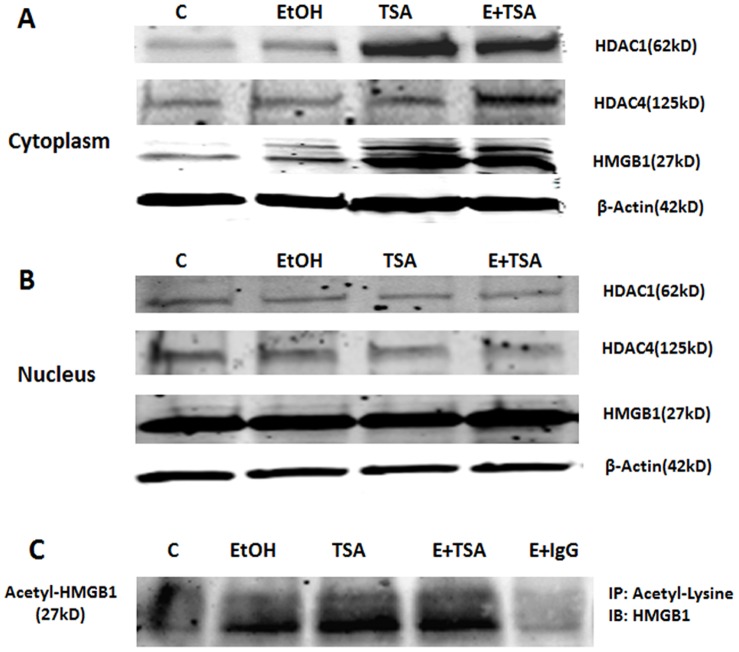
TSA-induced cytoplasmic mobilization of HDAC1 and 4 as well as HMGB1. A: Western blot analysis of HEC slice cytosolic protein extracts for HDAC1, HDAC4 and HMGB1 from a representative experiment with ethanol (100 mM) and TSA (1 µM). B: Western blot analysis of HEC slice nuclear protein extracts for HDAC1, HDAC4 and HMGB1 from the same experiments shown in A. C: Co-immunoprecipitation analysis of culture medium for acetylated HMGB1 level. Anti-rabbit IgG was used as a negative control.

**Figure 5:**
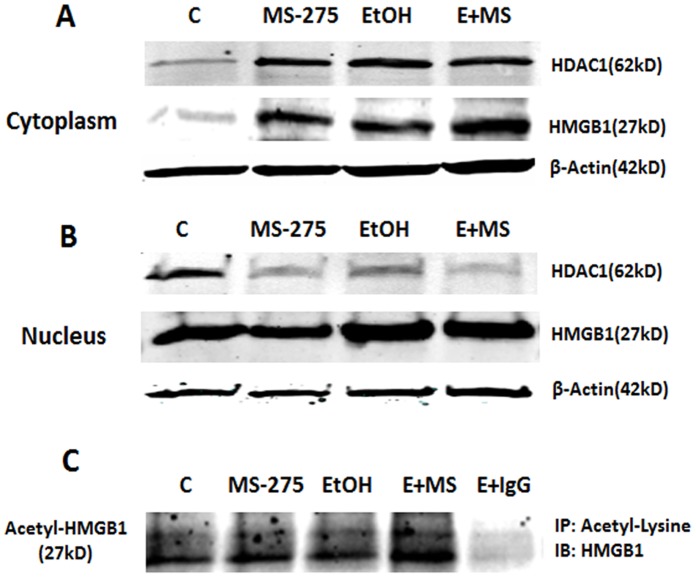
Inhibition of HDAC1 by MS-275 causing cytoplasmic mobilization of HDAC1 and HMGB1. A: Western blot analysis of HEC slice cytosolic protein extracts for HDAC1 and HMGB1 from a representative experiment with ethanol (100 mM) and MS-275 (5 µM). B: Western blot analysis of HEC slice nuclear protein extracts for HDAC1, HDAC4 and HMGB1 from the same experiments shown in A. C: Co-immunoprecipitation analysis of culture medium for acetylated HMGB1 level. Anti-rabbit IgG was used as a negative control.

HMGB1 and HDACs have been shown to shuttle from nuclear to cytosolic compartments in hepatocytes [18]. To determine if HDACs alter nuclear and cytosolic distribution in brain cells we treated HEC slices with TSA or MS-275 and separated cytosolic and nuclear fractions followed by Western blot analysis. We assessed nuclear-cytoplasmic changes in distribution of HMGB1 as well as HDAC1 and HDAC4. Controls find greater protein levels in HMGB1, HDAC1 and HDAC4 nuclear fractions than cytosolic fractions (Figure 4 and 5). Treatment of HEC slices with TSA, ethanol and combined ethanol+TSA increased HMGB1protein in cytoplasmic fractions (Figure 4A). Ethanol also slightly increased cytosolic HDAC1 and HDAC4. TSA markedly increased HDAC1 and HDAC4 in the cytosol, and combined ethanol-TSA increased cytosolic protein with clearly decreasing nuclear protein levels (Figure 4B). MS-275, a selective HDAC1 inhibitor, similarly increased cytosolic HMGB1 and HDAC1 (Figure 5A). These findings support the hypothesis that HDAC inhibitors and ethanol increase acetylation of HMGB1 leading to increased cytoplasmic HMGB1 and subsequent release. This hypothesis suggests released HMGB1 should be acetylated HMGB1. To assess acetylation of released HMGB1 we used co-immunoprecipitation (IP) of media with acetyl-lysine antibodies and then performed Western blots to determine levels of acetylated HMGB1. Treatment with ethanol, HDAC inhibitors TSA (Figure 4C) and MS-275 (Figure 5C), clearly increased acetylated HMGB1 level in media and combinations of ethanol with HDAC inhibitors, markedly increased acetylated HMGB1 release. These findings are consistent with HDAC inhibitors and ethanol increasing secretion of acetylated HMGB1.

### Ethanol Modulation of HDAC Activity

**Figure 6:**
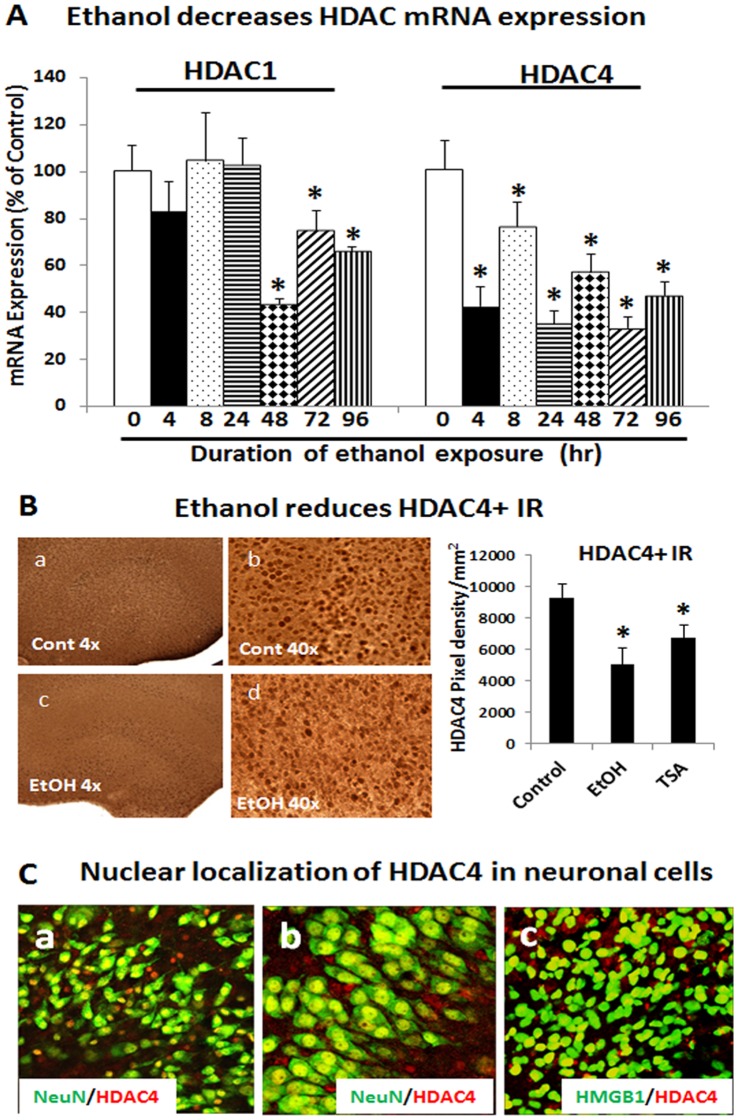
Effects of ethanol on HDAC mRNA and protein expression. A: Shown are mean ± SEM of HDAC1 and HDAC4 mRNA levels from a representative time course study. Ethanol exposure inhibits both HDAC1 and HDAC4 mRNA expression (*p<0.05 compared with control; n = 3). B: HDAC immunoreactivity (IR) was examined. Shown in bar graph (left) indicate both ethanol and TSA reduce HDAC4 IR in hippocampus (*p<0.05 compared with Control, n = 6). Representative images of HDAC4 IR were depicted from both control (a–b) and ethanol-treated (c–d) slices as indicated at 20x and 40x magnification. C: Representative confocal images of double immunofluorescent staining with HDAC4 (red) and neuronal marker NeuN (green) (a–b) and HMGB1 (c) indicating nuclear localization of HDAC4 in neuronal cells from control slices (a from dentate gyrus; b from hippocampal CA field; c from dentate gyrus). Original magnification 80x.

**Figure 7:**
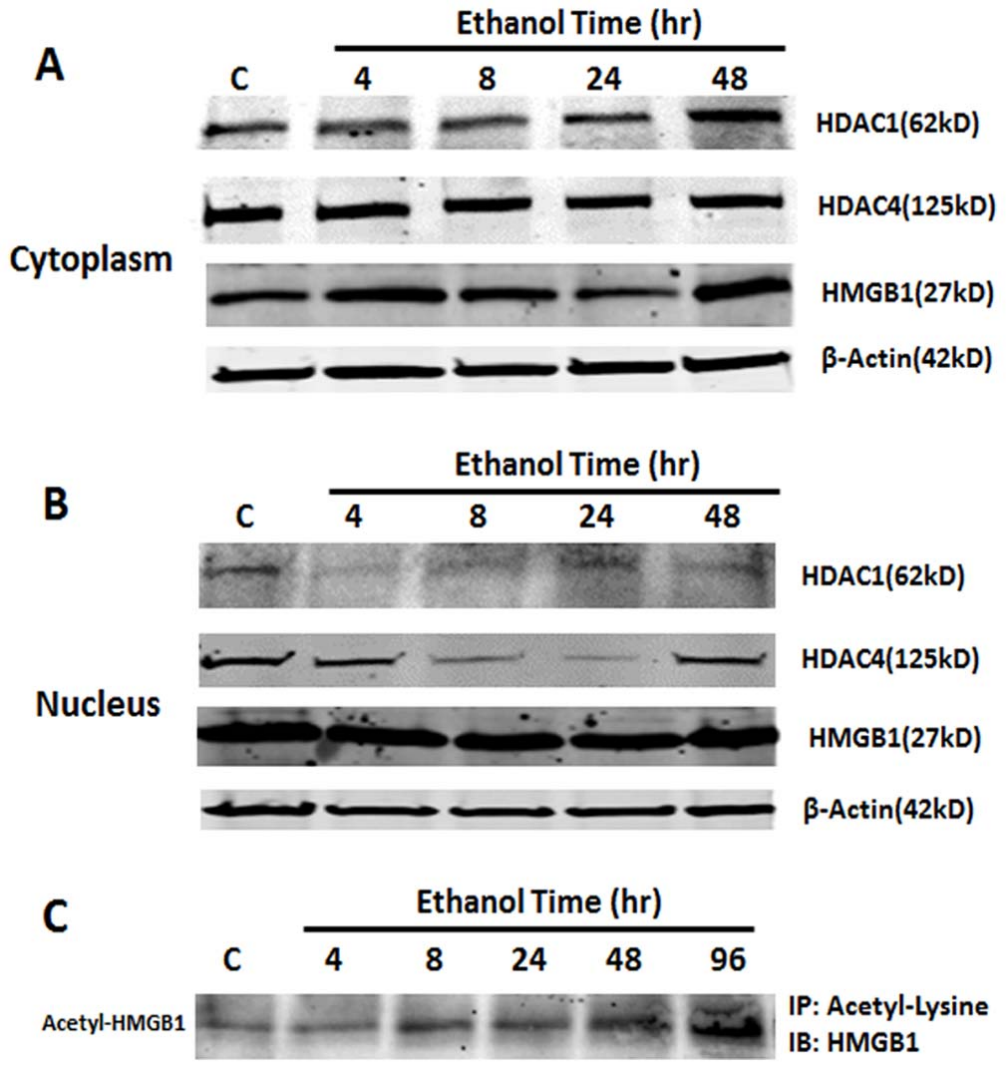
Western blot analysis of HDAC1 and HDAC4 as well as acetylated HMGB1. A: Western blot analysis of HEC slice cytosolic protein extracts for HDAC1, HDAC4 and HMGB1 from a representative ethanol time course experiment. B: Western blot analysis of HEC slice nuclear protein extracts for HDAC1, HDAC4 and HMGB1 from the same experiments shown in A. C: Co-immunoprecipitation analysis of culture medium for acetylated HMGB1 level. Anti-rabbit IgG was used as a negative control.

Ethanol treatment mimicked HDAC inhibitor treatment prompting experiments to examine the effects of ethanol on HDAC activity in HEC brain slice cultures. Ethanol treatment reduced HDAC mRNA (Figure 6-A). HDAC4 mRNA was decreased by about 50% after 4 hrs of ethanol treatment whereas HDAC1 was decreased after 48 hrs. Both remained suppressed for up to 96 hrs of ethanol treatment. Immunohistochemical analysis further confirmed that ethanol treatment significantly reduces HDAC4+IR (Figure 6-B) corresponding with the decrease in mRNA. Double immunofluorescent labeling reveals nuclear localization of HDAC4 in many NeuN+ neuronal cells and co-nuclear localization with HMGB1 in neurons (Figure 6-C). Separation of cytosolic and nuclear fractions after various time points of ethanol treatment finds increased cytosolic HDAC proteins coinciding with decreased nuclear HDAC level (Figure 7A–B). Changes in nuclear and cytosolic HDAC are apparent after a little as 4 hrs of ethanol treatment.

Consistent with ethanol disrupting HDAC activity and releasing acetylated HMGB1, ethanol treatment for as little as 4 hrs increases release of acetylated HMGB1 that progressively increases with time of ethanol treatment (Figure 7C). These results indicate that ethanol decreases HDACs activity and increases acetylated HMGB1 release, similar to TSA or MS-275, suggesting a possible involvement of HDAC activity in controlling HMGB1 release from neurons.

### Exogenous HMGB1 Induces Proinflammatory Cytokine Expression through TLR4 Activation

**Figure 8:**
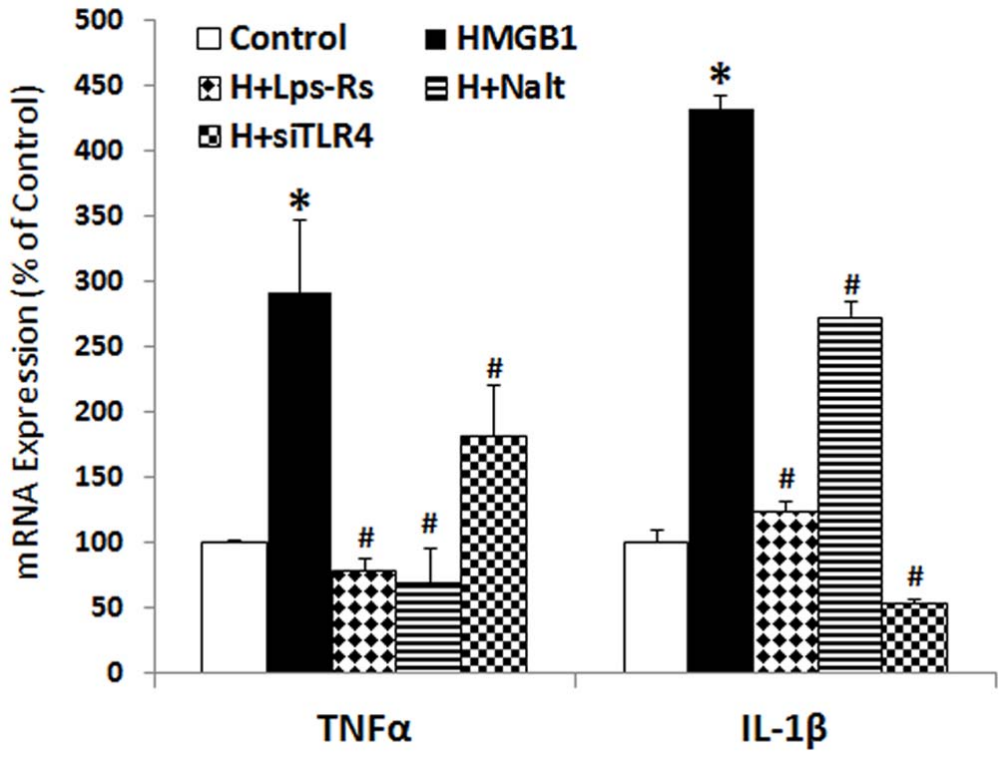
Exogenous HMGB1 induces expression of proinflammatory genes. HEC slices were treated with HMGB1 (500 ng/ml) for 96 hrs. and then harvested for detection of proinflammatory cytokine gene expression. Shown are mean ± SEM of cytokines TNFα and IL-β mRNA levels from a representative experiment. Treatment of HEC slices with exogenous HMGB1 induced significant increase in cytokine gene expression. Proinflammatory activity of HMGB1 is abolished by blockade of TLR4 with antagonists Lps-Rs and naltrexone or by knocking down TLR4 with specific siRNA (*p<0.01 compared with Control; #p<0.05 compared with HMGB1 group, n = 3).

To investigate the action of released HMGB1 by ethanol, we first performed a set of experiments to determine the proinflammatory activity of exogenous HMGB1. Treatment of HEC slices with exogenous HMGB1 increased proinflammatory cytokine TNFa and IL-1β mRNA level by several fold (Figure 8). The TLR4 antagonist, *Rhodobacter sphaeroides* LPS (Lps-Rs) [28] as well as TLR4 siRNA knockdown significantly reduces HMGB1 induction of proinflammatory cytokine genes. Interestingly, naltrexone (250 fM), the opiate antagonist recently found to block TLR4 receptors [29] also blocked HMGB1 induction of TNFα and IL-1β mRNA (Figure 8). These studies support the hypothesis that HMGB1 activates TLR4 receptors inducing TNFα and IL-1β mRNA.

### HMGB1 Mediates Ethanol Induction of Proinflammatory Genes through Microglial TLR4

**Figure 9:**
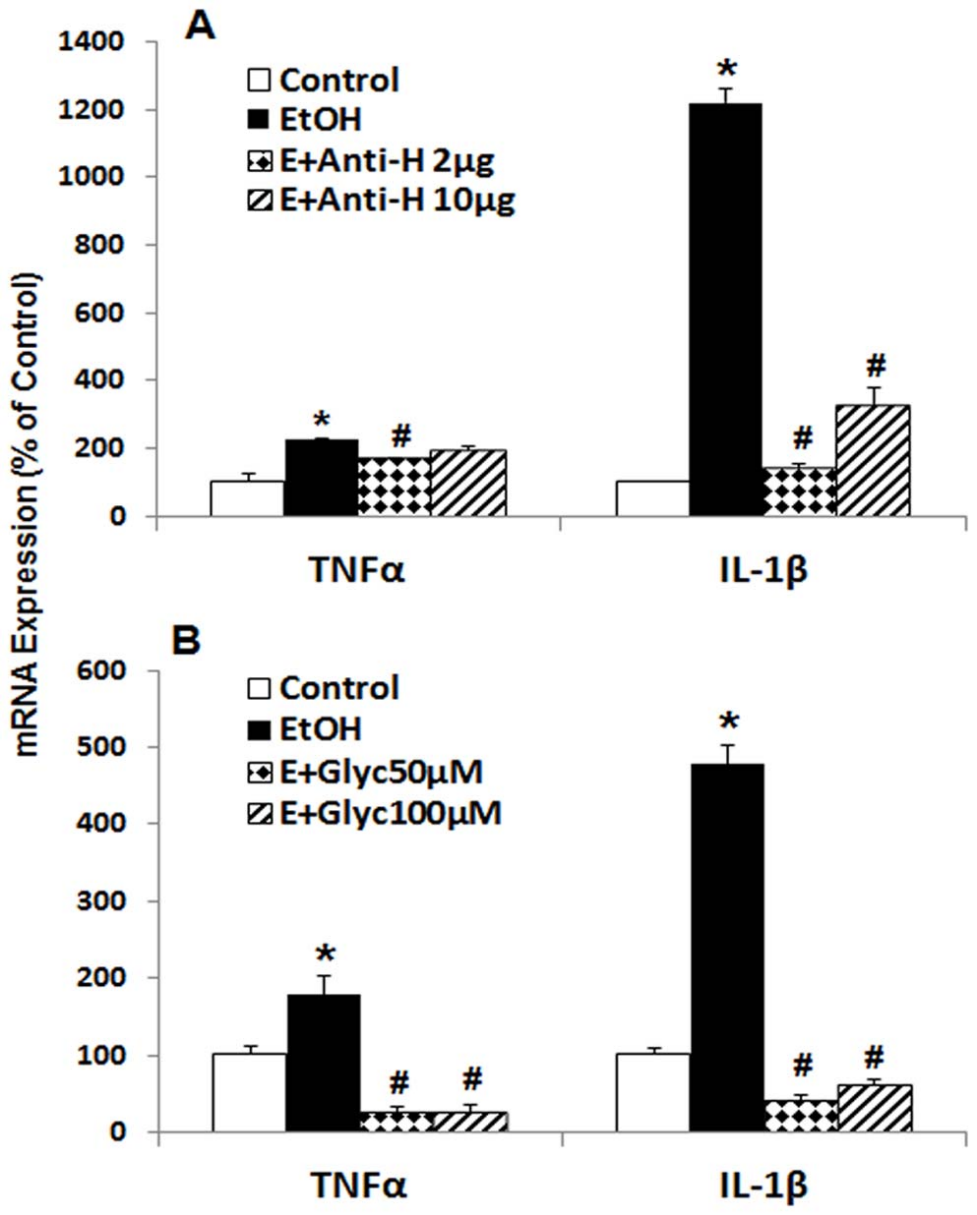
HMGB1-mediated ethanol neuroimmune activation. A: HEC slices were exposed to ethanol (100 mM) for 4 days in the absence or presence of anti-HMGB1 neutralizing antibody. Chicken IgY (10 µg/ml) was used as control antibody for HMGB1 neutralization. Shown are mean ± SEM of proinflammatory cytokine TNFα and IL-1β mRNA levels, relative to chicken IgY-treated control. Ethanol induction of TNFα and IL-1β mRNA is blocked by HMGB1 neutralizing antibody (*p<0.01 compared to Control; #*p*<0.05 compared with EtOH; n = 3). B: HMGB1 inhibitor glycyrrhizin (Glyc) was applied to the cultures during ethanol (100 mM) treatment for 4 days. Shown are mean ± SEM of proinflammatory cytokine TNFα and IL-1β mRNA levels from a representative experiment. Ethanol induction of TNFα and IL-1β mRNA is completely blocked by glycyrrhizin (*p<0.001 compared to Control; #*p*<0.0001 compared with EtOH; n = 3). The experiments were repeated at least once with the similar design and results.

Previous studies have suggested that brain HMGB1, TLR4 receptors and microglia are critical components of ethanol induction of proinflammatory genes [12]. To explore the role of ethanol released HMGB1 in induction of proinflammatory genes we used HMGB1 neutralizing antibodies and inhibitors. Ethanol-increased mRNA levels of both TNFα and IL-1β were blunted by the addition of HMGB1 neutralizing antibodies (Figure 9A). Glycyrrhizin is a natural product from licorice with anti-inflammatory properties linked to binding and inactivation of HMGB1 [30]. Glycyrrhizin addition to slices reduced ethanol induction of TNFα and IL-1β (Figure 9B). Ethanol induction of IL-1β varied between 5 and 12 fold in these two experiments. This pronounced induction by ethanol was reduced to near or below baseline levels by HMGB1 neutralizing antibodies and inhibitor glycyrrhizin respectively, suggesting released HMGB1 mediates ethanol inflammatory response.

**Figure 10:**
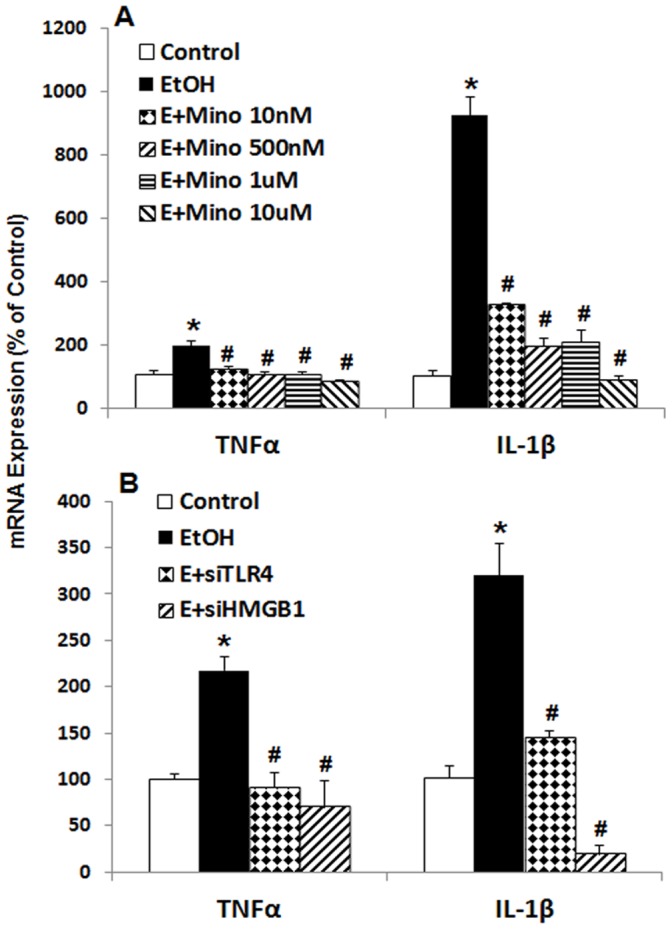
Microglial TLR4 plays a critical role in HMGB1-mediated ethanol neuroimmune activation. A: Minocycline, known to inhibit microglial activation, was applied to HEC slices at different concentrations during ethanol exposure (100 mM, 4 days). Shown are mean ± SEM of proinflammatory cytokine TNFα and IL-1β mRNA levels. Blocking microglial activation by minocycline blunt ethanol induction of cytokine TNFα and IL-1β mRNA (*p<0.01 compared with Control; #p<0.01 comparing to EtOH; n = 3). The experiments were repeated twice with similar design and results. B: Knocking down microglial TLR4 or neuronal HMGB1 with specific siRNA abolished ethanol neuroimmune activation. Shown are mean ± SEM of proinflammatory cytokine TNFα and IL-1β mRNA levels relative to control group treated with negative control siRNA (*p<0.001 compared with Control; #p<0.0001 compared with EtOH; n = 3). The experiments were repeated with similar designs and results.

**Figure 11:**
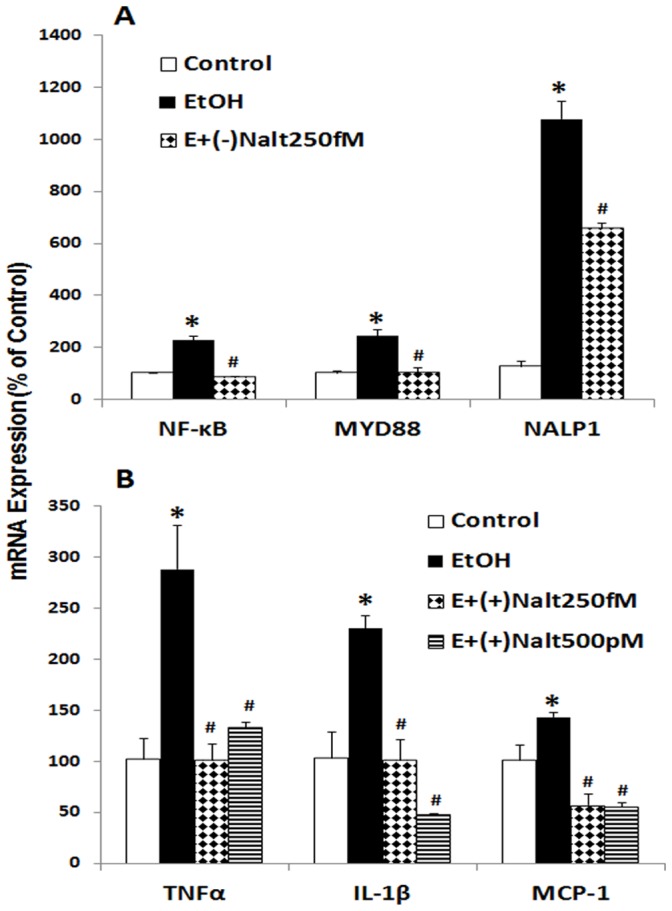
TLR4 antagonist naltrexone blocks ethanol neuroimmune activation. A: Ethanol (100 mM, 4 days) induction of proinflammatory signaling molecules were significantly blocked by TLR4 antagonist naltrexone (minor form, 250 fM). Shown are mean ± SEM of mRNA levels relative to control (*p<0.001 compared with Control; #p<0.01 compared with Ethanol; n = 3). B: Ethanol induction of TNFα and IL-1β mRNA is completely blocked by naltrexone (plus form, 250 pM) (*p<0.001 compared to Control; #*p*<0.0001 compared with EtOH; n = 3).

Previous studies have suggested TLR4 receptors on microglia are critical to ethanol induction of proinflammatory genes [2], [31], [32]. In experiments investigating ethanol induced sensitization of TLR agonist activation of brain microglia and neurotoxicity in mice, we found minocycline, an inhibitor of microglial activation [33], [34], reduces both morphological microglial activation and markers of neuronal death [35]. We report here that increasing concentrations of minocycline blunted ethanol induction of TNFα and IL-1β in a concentration dependent manner (Figure 10A). Further, gene knockdown using HMGB1siRNA or TLR4siRNA blocked ethanol induction of TNFα and IL-1β mRNA (Figure 10B). We also tested naltrexone which has recently been found to interact with the TLR4 receptor as well as the opiate receptor [29]. We tested both optical isomers of naltrexone since the (−) isomer is both an opiate receptor antagonist and a TLR4 antagonist whereas the (+) isomer is only a TLR4 antagonist and not an opiate antagonist. Both (−) and (+) naltrexone effectively blocked ethanol induction of proinflammatory genes (Figure 11A and B). These studies are consistent with previous studies that inflammatory activity of ethanol is medicated by HMGB1 released from neuronal cells [36]. Minocycline antagonism suggests ethanol released HMGB1 from neurons may initiate proinflammatory responses through activation of microglia TLR4, however, the mechanisms of minocycline inhibition of microglial activation are poorly understood and linked to inhibition of p38 kinase activation [34]. Some neurons express p38-kinase [37] and it is possible that minocycline also impacts neuronal neuroimmune signaling. Additional studies are needed to clearly define the brain cell types responding to HMGB1 release. These studies are consistent with previous studies that inflammatory activity of ethanol is medicated by HMGB1 released from neuronal cells. Minocycline antagonism suggests ethanol released HMGB1 from neurons may initiate inflammatory response through activation of microglia TLR4.

## Discussion

**Figure 12:**
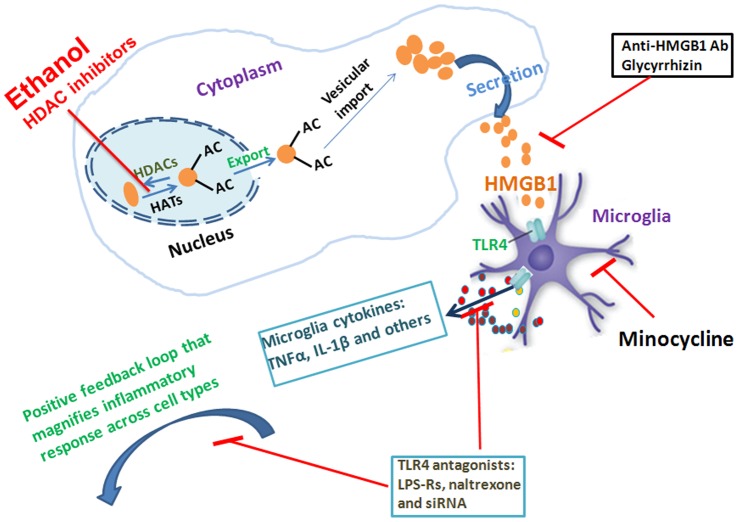
Schematic of ethanol-induced release of neuronal HMGB1 activating microglial TLR4 and creating a positive feedback loop that amplifies innate immune cascades. Ethanol exposure decreases neuronal HDACs activity causing increased acetylation of HMGB1. Acetylated HMGB1 is mobilized into cytosolic compartment and subsequently secreted into extracellular space. Extracellular HMGB1 activates TLR4 in microglia to initiate neuron-glia neuroimmune communication by producing a wide array of proinflammatory cytokines or mediators such as TNFα, IL-1β, iNOs and MCP-1. Activated microglia may also induce expression and release of HMGB1 [56], [57]. Proinflammatory cytokines and extracellular HMGB1 create a positive feedback loop that amplifies neuroimmune responses with engagements of other TLRs and RAGE as well as individual cytokine receptors across cell types. In addition, extracellular HMGB1 can exert potent proinflammatory activity by forming complexes with proinflammatory cytokines TNFα and IL-1β (unpublished data). Targeting multiple sites involving in HMGB1-TLR4 signaling may prove to be effective therapeutic approaches for combating brain neuroimmune activation associated with alcoholism and drug addiction [1], [47].

HMGB1 is a ubiquitous protein that is highly expressed in neurons [12], [22]. We report here that ethanol and HDAC inhibitors release acetylated HMGB1 from neuronal cells in HEC slice cultures. It is possible that osmotic effects of added ethanol contribute to HMGB1 release, although visual inspection of ethanol treated neurons in Fig. 2 do not show swelling and acetylated HMGB1 is released supporting an active mechanisms of HMGB1 release, we cannot rule out an osmotic effect. Our data further indicates that ethanol-induced expression of proinflammatory cytokines TNFα and IL-1β was blunted by the blockade of both HMGB1 using HMGB1 neutralizing antibodies and inhibitors and TLR4 using TLR4 siRNA and TLR4 antagonists including Lps-Rs and naltrexone, consistent with HMGB1 release activating TLR4 receptors. Consistent with our finding in HEC slices, HMGB1 treatment of neuronal-astrocyte cultures induces HMGB1 mRNA as well as proinflammatory cytokines without increasing PI assessed cell death, although HMGB1 did sensitize to glutamate excitotoxicity [38]. Our findings for the first time indicate that neurons release HMGB1 through an active mechanism related to changes in status of HMGB1 acetylation that is regulated by HDACs activity, consistent with studies in monocytes and hepatocytes that find HMGB1 acetylation regulates movement from the nucleus to cytoplasm and subsequent release [18], [19]. HDACs regulation of HMGB1 acetylation may represent an important regulator of neuronal HMGB1 release. These novel findings suggest active release of HMGB1 being an endogenous TLR4 ligand contributing to “sterile” neuroimmune activation in brain by ethanol (Figure 12).

HMGB1, TLR4 receptors and/or neuroimmune activation are linked to many brain diseases including depression [39] and neurodegeneration [40]. For example, in animal models of stroke, HMGB1 is released rapidly before pronounced cell death [41] and treatment with neutralizing HMGB1 antibodies or HMGB1 inhibitor glycyrrhizin reduces ischemia-induced neuroimmune gene induction and brain damage [42], [43], [44] as well as spinal cord damage [44]. Our findings indicate HMGB1 release activates TLR4 inducing proinflammatory cytokines TNFα and IL-1β. Since HMGB1 is predominantly localized in neuronal cells, our studies suggest ethanol and HDAC inhibitors release HMGB1 from neurons. Recent studies have found that neuronal excitation releases HMGB1 activating glial TLR4 receptors that contribute to the generation and perpetuation of epileptic seizures [23]. Our findings extend these studies to ethanol release of HMGB1 and may suggest a key role of HMGB1 in persistent and progressive brain neuroimmune activation (Figure 12). We have previously found that brain neuroimmune activation persists for long periods that contribute to neurodegeneration [10], [12], [31], [35]. It is conceivable that neurons release danger signal HMGB1 in response to stimuli and HMGB1 initiates crosstalk of neuroimmune signaling between neurons and glia. The findings reported here support the notion that positive feedback loops of neuroimmune activation in brain may require HMGB1 activation of microglial TLR4 resulting in production of microglia-released proinflammatory mediators such as TNFa and IL-1β, which further augments release of HMGB1 from neurons and other cells (Figure 12). This is consistent with studies finding that mice lacking TLR4 are protected against brain insults including models of ischemia, trauma, genetic neurodegeneration and epilepsy as well as alcohol and toxin induced neuropathology [2], [40], [45].

Accumulating evidence from a broad range of studies supports neuroimmune activation as important contributing factors to alcoholism. Indeed, study of post-mortem human alcoholic brain has found increased microglial markers and the proinflammatory molecules [3], [4] as well as increased expression of HMGB1 and TLR receptors [12]. Chronic ethanol treatment of mice also increases brain proinflammatory gene expression [5] as well as HMGB1 and TLR4 expression [12]. Mice with genetic predisposition for high alcohol consumption as well as human alcoholics show changes in brain expression of genes related to immune signaling [46]. Studies of the genetic determinants of high voluntary alcohol drinking across multiple mouse strains have found NF-κB and proinflammatory cytokine signaling genes among the molecular determinants [8]. Human genetic association studies find multiple alleles of neuroimmune genes, including NF-κB are associated with alcoholism [1], [47]. Mice lacking neuroimmune genes including genes important for TLR4 signaling, e.g. CD14, have a lower preference and drink less alcohol [46]. Recent studies indicate that mice treated with LPS inducing brain neuroimmune gene induction drink more alcohol than controls for long periods [9]. Transgenic mice lacking TLR4 receptors do not show ethanol induced activation of proinflammatory genes in astrocytes [48] or microglial cultures [32]. Chronic ethanol treatment of mice lacking TLR4 receptors similarly finds reduction of ethanol-induced brain proinflammatory gene expression, neurodegeneration, dopamine alterations and alcoholic behavioral pathologies [6], [49] and damage to myelin [49]. In previous studies we found ethanol treatment of HEC slice cultures increased proinflammatory transcription factor NF-κB-DNA binding and expression of NF-κB target genes TNFα, IL-1β, MCP1 and iNOS [7], [50]. The present study for the first time identifies that endogenous danger signal HMGB1 can be mobilized from neurons, translocate into cytoplasmic compartments and subsequently released into extracellular space upon ethanol exposure. Neuronal HMGB1-microglia TLR4 signaling may be the centerpiece of innate immunity in brain in response to alcohol consumption. We report here that ethanol responses can be effectively attenuated by either HMGB1 antagonism or TLR4 antagonism. It is worth mentioning here that naltrexone, an opiate receptor antagonist recently found to be a TLR4 antagonist [29], blocks ethanol-HMGB1 activation of proinflammatory signaling. Naltrexone is used as a pharmacotherapy for alcoholism [51] and brain neuroimmune activation has been suggested to contribute to addiction [52]. Naltrexone could alter neuroimmune responses through opiate antagonism and/or through TLR4 receptor antagonism. Together, these studies are consistent with HMGB1-TLR4 activation of neuroimmune signaling as contributing factors to alcoholism and alcoholic neuropathology. Our findings may provide insight into existing therapeutics and/or may represent new treatment targets for alcoholism.

We report here that HDAC inhibitors release acetylated HMGB1 from HEC brain slice cultures and increase cytosolic HMGB1, HDAC1 and HDAC4. Ethanol also released acetylated HMGB1 and increased cytosolic HMGB1, HDAC1 and HDAC4. Previous studies have shown ethanol treatment of mice decreases brain HDAC activity [53]. Other studies in rats also report increases in brain histone acetylation associated with increased histone acetyltransferase activity that is potentiated by HDAC inhibitors [54]. Ethanol inhibition of HDAC in amygdala increases histone acetylation associated with the anxiolytic actions of ethanol [55]. We found that ethanol treatment decreased HDAC1/4 mRNA levels acompanying with increase in release of acetylated HMGB1. Similarly, studies in hepatocytes and mononcytes demonstrate nuclear HDAC activity modulates HMGB1 acetylation and cytoplasmic translocation and subsequently release [18], [19]. Together these studies support the hypothesis that alterations in acetylase enzymes regulate HMGB1 release and neuroimmune activaton in brain.

## Conclusions

In summary, we find that HMGB1 is highly expressed in neurons in HEC cultures and ethanol decreases HDAC activity triggering releases of neuronal HMGB1 (Figure 12). Extracellular HMGB1 subsequently activates microglial TLR4 leading to production of proinflammatory cytokines TNFα and IL-1β. Targeting either HMGB1 using HMGB1 neutralizing antibodies and inhibitor glycyrrhizin or TLR4 using TLR4 antagonists, TLR4 siRNA can effectively attenuate ethanol inflammatory response (Figure 12). These findings suggest multiple new potential targets to block brain neuroimmune activation, alcoholic neurodegeneration and alcoholism.
